# Mechanisms underlying rhizosheath dynamics in *Kengyilia hirsuta* in response to alternating drought and rewatering

**DOI:** 10.1038/s41598-026-49036-7

**Published:** 2026-04-24

**Authors:** Yutao Yuan, Li Wu, Jianhong Zhang, Chen Chen, Qingping Zhou, Youjun Chen

**Affiliations:** 1 Bailie Vocational College, Zhangye, 734000 China; 2 College of Grassland Resources, Southwest Minzu University, Chengdu, 610041 China; 3 Academy of Animal Science and Veterinary Medicine of Qinghai University, Xining, 810016 China

**Keywords:** Drought- rehydration, *Kengyilia hirsuta*, Rhizosheath, Root architecture, AM fungi, Ecology, Ecology, Plant sciences

## Abstract

Under increasing frequency of extreme climate events, plant adaptation to alternating drought–rewatering stress is critical. *Kengyilia hirsuta*, a pioneer forage grass in alpine desert ecosystems, relies on rhizosheath formation for drought resistance. This study conducted indoor pot experiments with six water treatments: three drought–rewatering cycles (W1–W3, re‑watered to 10%, 25%, and 40% of field capacity, FC) and three sustained drought levels (W4–W6, maintained at 10%, 25%, and 40% FC). Root architecture, biomass allocation, arbuscular mycorrhizal fungi (AMF) colonization, and rhizosheath formation were examined over three successive 7‑day periods (T1–T3). Results revealed dynamic responses of rhizosheath accumulation to water regimes: maintained 25% FC (W5) significantly promoted rhizosheath biomass, maintained 40% FC (W6) enhanced early‑stage development, and re‑watering to 10% FC (W1) boosted later‑stage formation. AMF colonization increased progressively, with total colonization rising from 41.51% at T1 (day 7) to 61.40% at T3 (day 21). The W5 treatment consistently exhibited the highest vesicle, arbuscule, and hyphal colonization, along with increased soil spore density and hyphal density by T3. Root morphological traits—including tip number, volume, hair length, and hair density—also peaked under W5. Structural equation modelling identified AMF colonization (total effect: –0.90) and root hair traits (total effect: +0.80) as pivotal regulators of rhizosheath formation. This negative total effect of AMF colonization does not indicate overall inhibition, but rather reflects the feedback regulation intensity mediated by microbial competition and the carbon allocation trade-off within the plant-fungal symbiosis under resource-limited conditions. These factors interact through biomass allocation, root architecture, and soil microenvironment, forming a multidimensional adaptive network. These findings elucidate the ecophysiological mechanisms of plant–AMF collaboration in rhizosheath formation under water fluctuation, supporting the selection of stress‑tolerant grasses for restoring desertified grasslands.

**Supplementary Information:**

The online version contains supplementary material available at 10.1038/s41598-026-49036-7.

## Introduction

**Fig. 1:**
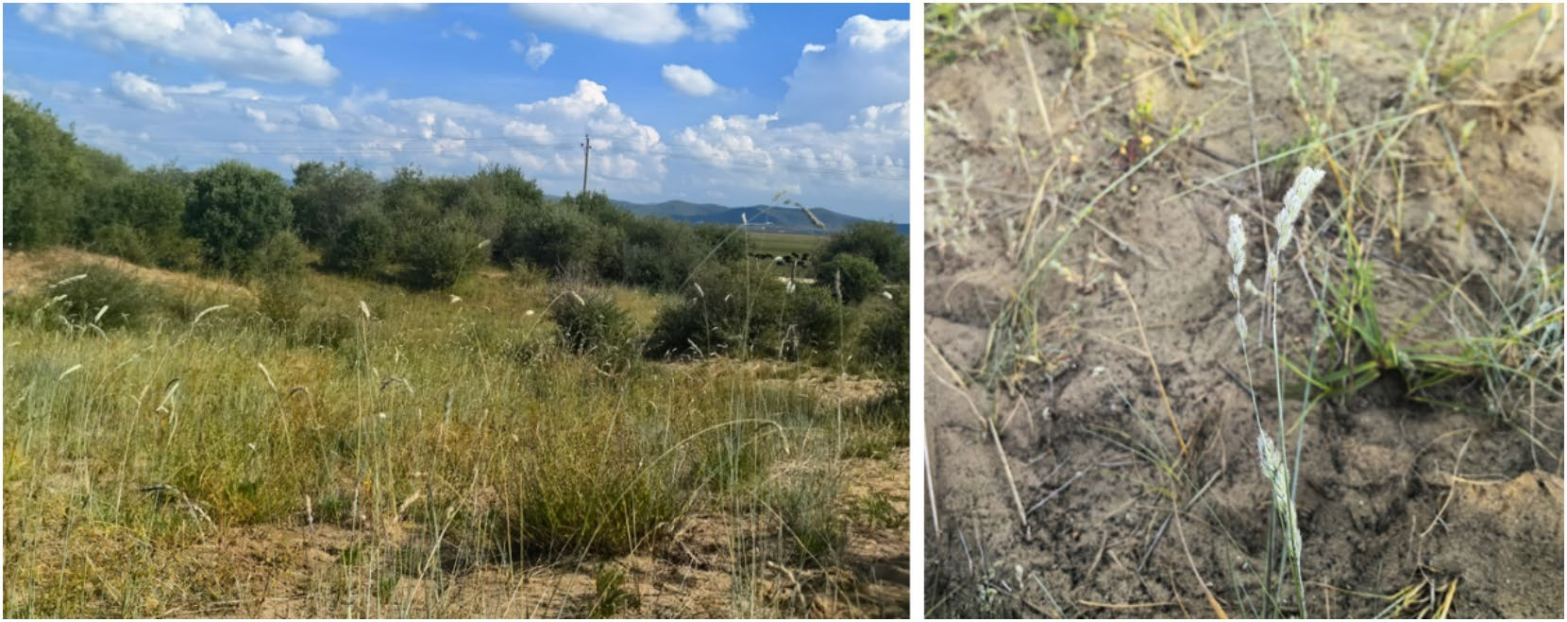
Experimental materials: habitat, plant morphology of *K. hirsuta.*

*Kengyilia hirsuta* is a perennial hexaploid species within the Poaceae family, characterised by long, yellow-brown, stiff hairs on the lemmas^1^. As a pioneer forage grass and a source of stress-tolerant germplasm, it plays a vital role in combating desertification and in alpine forage breeding on the Qinghai–Tibet Plateau^2,3^. This species forms a highly specialised rhizosheath—a sheath-like structure in which root exudates bind soil particles (Mo et al.,^4^). Functioning as a critical interface between roots and soil, the rhizosheath offers physical protection to roots, regulates water dynamics, and facilitates microbial interactions^5,6^. Under drought stress, it enhances plant tolerance by improving water uptake^6,7^, promoting the growth of beneficial microorganisms^8^, and strengthening soil aggregation^9^. Consistent with these roles, our field observations revealed pronounced rhizosheath development in *K. hirsuta* under arid conditions, supporting the hypothesis that this trait has evolved in response to long-term water scarcity (Fig. 1).

Drought stress significantly inhibits root development and biomass accumulation in plants, largely due to disruptions in water metabolism, photosynthetic efficiency, and cellular homeostasis^10^. To adapt to arid environments, plants have evolved diverse strategies, among which rhizosheath formation represents a key mechanism for drought tolerance. In particular, roots promote rhizosheath development through architectural modifications and the secretion of mucilaginous substances^11^. This enhances water-use efficiency—a phenomenon documented in crops such as rice (*Oryza sativa*) and chickpeas (*Cicer arietinum*)^8,12^. Moreover, the rhizosheath serves as an important ecological niche for functional microorganisms, selectively enriching drought-resistant taxa such as *Massilia*, *Nocardioides*, and arbuscular mycorrhizal fungi (AMF)^12^. AMF, in particular, exhibit synergistic effects with their host plants by improving water uptake and nutrient translocation^13,14^. Although rehydration following drought has been shown to stimulate compensatory growth in plants^15^, its influence on the formation and restructuring of rhizosheaths and their associated microbial communities remains unclear.

Current understanding of the mechanisms by which *K. hirsuta* responds to drought remains limited. Several key knowledge gaps persist: first, the dynamics of rhizosheath formation under alternating drought–rehydration conditions and its feedback relationship with AMF; second, the interplay between root architecture, biomass allocation, and rhizosheath development; and third, the mechanisms underlying rhizosheath-mediated plant–microbe synergism in drought resistance. This study employs graded drought and rehydration treatments to systematically analyse the relationships among rhizosheath formation, AMF colonisation, root phenotype, and biomass allocation, with the aim of addressing these research gaps. The ultimate objectives are to clarify the functional role of the rhizosheath in drought adaptation and to establish a theoretical foundation for the restoration of desertified grasslands.

## Materials and methods

### Plant materials and growth conditions

Seeds of *K. hirsuta* were provided by the College of Grassland Resources, Southwest Minzu University. These seeds were collected from WaQie Town, Hongyuan County (33.18° N, 102.62° E, altitude 3490 m), Aba Prefecture, Sichuan Province, China in September 2022, with permission granted by the Forestry and Grassland Bureau of Hongyuan County (Fig. 1). The plant species was authoritatively identified by Professor Junliang Yang from the College of Life Sciences, Sichuan Agricultural University. A voucher specimen (voucher number: SAUT 201402232) has been deposited in the Herbarium of the Triticeae Research Institute, Sichuan Agricultural University (SAUT).

The growth substrate consisted of sandy soil with the following physicochemical properties: pH 7.69, organic carbon 6.3 g/kg, total phosphorus 0.35 g/kg, nitrate nitrogen 6.68 mg/kg, ammonium nitrogen 6.69 mg/kg, and a maximum field capacity of 23%. Before use, the soil was air-dried naturally and sieved through a 4 mm mesh.

### Experimental design

Mature, plump seeds of *K. hirsuta* with uniform morphology were selected. To break dormancy, seeds were stratified at 4 °C for 48 hours, then surface-sterilized with 75% (v/v) ethanol for 30 seconds and rinsed three times with sterile water. The sterilised seeds were placed on moist filter paper in Petri dishes and germinated in a controlled climate chamber (photoperiod 16 h/8 h, day/night temperature 24°C/20°C, light intensity 5000 lx, relative humidity 50%–60%). When the radicles reached approximately 3 cm in length, uniformly grown seedlings were selected and transplanted into pots (10 cm × 7 cm × 8.5 cm). Each pot, filled with 0.5 kg of prepared sandy soil, contained five seedlings, for a total of 120 pots.

The drought stress gradients in this experiment were established based on a preliminary study on the root physiology of *K. hirsuta*. In that study, we found that the rhizosheath weight exhibited an initial increase followed by a decrease as soil moisture rose from 10% to 50% of field capacity (FC). This nonlinear response indicated that both severe drought and near-optimal moisture conditions could alter root adaptive strategies, providing a physiological basis for defining the stress gradients in the present study^16,17^. Therefore, six treatments were designed to encompass both cyclic and sustained drought stresses within this critical moisture range. Water treatments commenced at the three-leaf stage. The Drought-Re-watering Cycle groups (7-day cycle) included: W1 (re-watered to 10% FC), W2 (re-watered to 25% FC), and W3 (re-watered to 40% FC). The Continuous Drought groups (maintained at constant daily moisture levels) included: W4 (maintained at 10% FC), W5 (maintained at 25% FC), and W6 (maintained at 40% FC).

A homogeneous sandy soil was used for the experiment. The field capacity was determined gravimetrically. Briefly, soil samples from the same batch were saturated and allowed to drain freely for 48 hours under conditions preventing evaporation. The soil was then oven-dried at 105 °C for 24 hours. The weights of wet soil after drainage and dry soil were measured separately, and the soil gravimetric water content (g water per g dry soil) calculated therefrom corresponds to the field capacity^18^.

For each pot, the target soil water mass was calculated as: (Mass of dry soil per pot × FC value) × target percentage (e.g., 10%, 25%, or 40%). Throughout the 21-day experimental period, the soil water content was maintained within ±2% of the target value using the daily weighing method. This involved daily measurement of the total pot weight (pot + soil + plant). The current soil water mass was derived by subtracting the known tare weight of the pot and the estimated plant biomass increment. The daily irrigation amount was then determined based on the difference between the current and target soil water mass. The entire experiment lasted 21 days, with corresponding growth indices measured at 7-day intervals (denoted as T1, T2, and T3, respectively). Four biological replicates were used for each treatment.

### Determination indicators and methods

#### Quantitative of rhizosheath

Photograph the rhizosheath under different treatments using a digital camera, with three shoots per treatment. Rhizosheath biomass was assessed using a combined mechanical vibration–ultrasonic separation approach. The whole plant was carefully excavated, and loose soil was removed by gentle shaking while preserving the intact rhizosheath. After root excision, the fresh weight of the root–rhizosheath complex (X1) was recorded. Roots were then ultrasonicated (40 kHz, 30 min) in deionised water to detach the rhizosheath completely. The roots were blot-dried and reweighed (X2), and rhizosheath biomass was calculated as the difference:

Rhizosheath weight (mg/cm) = (X1 – X2)/total root length.

Total root length was measured using a root scanner (EPSON Expression 12000XL, 600 dpi) and analysed with *WinRHIZO Pro* software (Version 2017a; https://www.regentinstruments.com/).

### Analysis of AM fungal colonization

#### Colonization rate

Root segments (1 cm) were cleared in 10% KOH at 90 °C for 90 min, acidified in 2% HCl, and stained with 0.05% Trypan blue in a lactic acid–glycerol solution at 90 °C for 30 min. After destaining in lactic acid–glycerol for 72 h, segments were mounted on slides and examined under an OLYMPUS BX53 microscope. Colonisation by vesicles, arbuscules, and hyphae was assessed using the grid intersection method across 400 fields per treatment. Colonisation rates were calculated as follows:

Vesicle colonisation rate = (Number of intersections with vesicles/Total intersections) × 100%

Arbuscule colonisation rate = (Number of intersections with arbuscules/Total intersections) × 100%

Hyphal colonisation rate = (Number of intersections with hyphae/Total intersections) × 100%

Total colonisation rate = [(Total intersections – No-colonised intersections)/Total intersections] × 100%

#### Hyphal density

Soil samples from the rhizosheath (after gently shaking off the root system, the soil adhering closely to the surface of the roots) and rhizosphere (Soil within a range of 2–5 mm from the root system) were collected separately. After air-drying and sieving, 5.0 g of each sample was mixed with deionized water to prepare a soil suspension. The suspension was successively passed through 20-mesh and 400-mesh sieves. The material retained on the 400-mesh sieve was collected, centrifuged, and the supernatant was filtered through a 0.45 μm membrane under vacuum. The membrane was stained with trypan blue and observed under an optical microscope. Twenty-five fields of view were randomly selected, and the grid-line intersection method was applied to count the intersections between hyphae and grid lines. Hyphae were distinguished based on morphological characteristics: arbuscular mycorrhizal fungi (AMF) hyphae were identified as aseptate, highly branched, and evenly stained, whereas non-AMF hyphae were typically septate. The following formula was used to determine AMF hyphal density and length^19^.

Hyphal length (m) = (11/14 × Number of intersections × Grid cell length × Membrane area × Dilution factor)/(25 × Grid area)

Hyphal density (m/g) = Hyphal length (m)/Soil mass (g)

#### Spore density

Spores were extracted from 10 g soil by wet sieving and enriched via sucrose gradient centrifugation (45% solution, 3,000 × g, 3 min). Collected spores were transferred to a Petri dish and counted under a Nikon SMZ25 stereomicroscope. Results are expressed as spores per gram of dry soil (spores/g).

#### Root phenotype analysis

Overall root architecture parameters—including total root length, surface area, volume, diameter, tip count, and branch number—were analysed using washed roots scanned at 600 dpi (Epson Expression 12000XL) and processed with *WinRHIZO Pro*. For root hair characterisation, segments taken 3–5 cm behind the root tip were mounted on slides, and the length of ten fully expanded root hairs per segment was measured using a Nikon Eclipse Ni phase-contrast microscope. Root hair density per unit length (root hairs/mm) was quantified with ImageJ 1.53s.

#### Determination of biomass and root-shoot ratio

Plants were separated into shoot and root parts. After cleaning, the fresh weight of each part was recorded. To determine biomass, the samples were first oven-dried at 105 °C for 10 min to terminate biological processes and then dried at 80 °C to constant weight before measuring the dry weight. The root-shoot ratio was calculated as the root dry weight divided by the shoot dry weight.

#### Data processing

Data were standardised and organised in Excel 2021, with outliers removed by Grubbs’ test. One-way ANOVA was performed in *R 4.2.1*, followed by LSD multiple comparisons (α = 0.05). Principal component analysis (PCA) was conducted using the vegan package, and Mantel tests (*ape package*) were used to assess associations between environmental factors and phenotypic traits. Figures were prepared with Origin 2021.

## Results and analysis

### Dynamic response characteristics of rhizosheath formation

#### Rhizosheath formation responds non-linearly to water stress

**Fig. 2:**
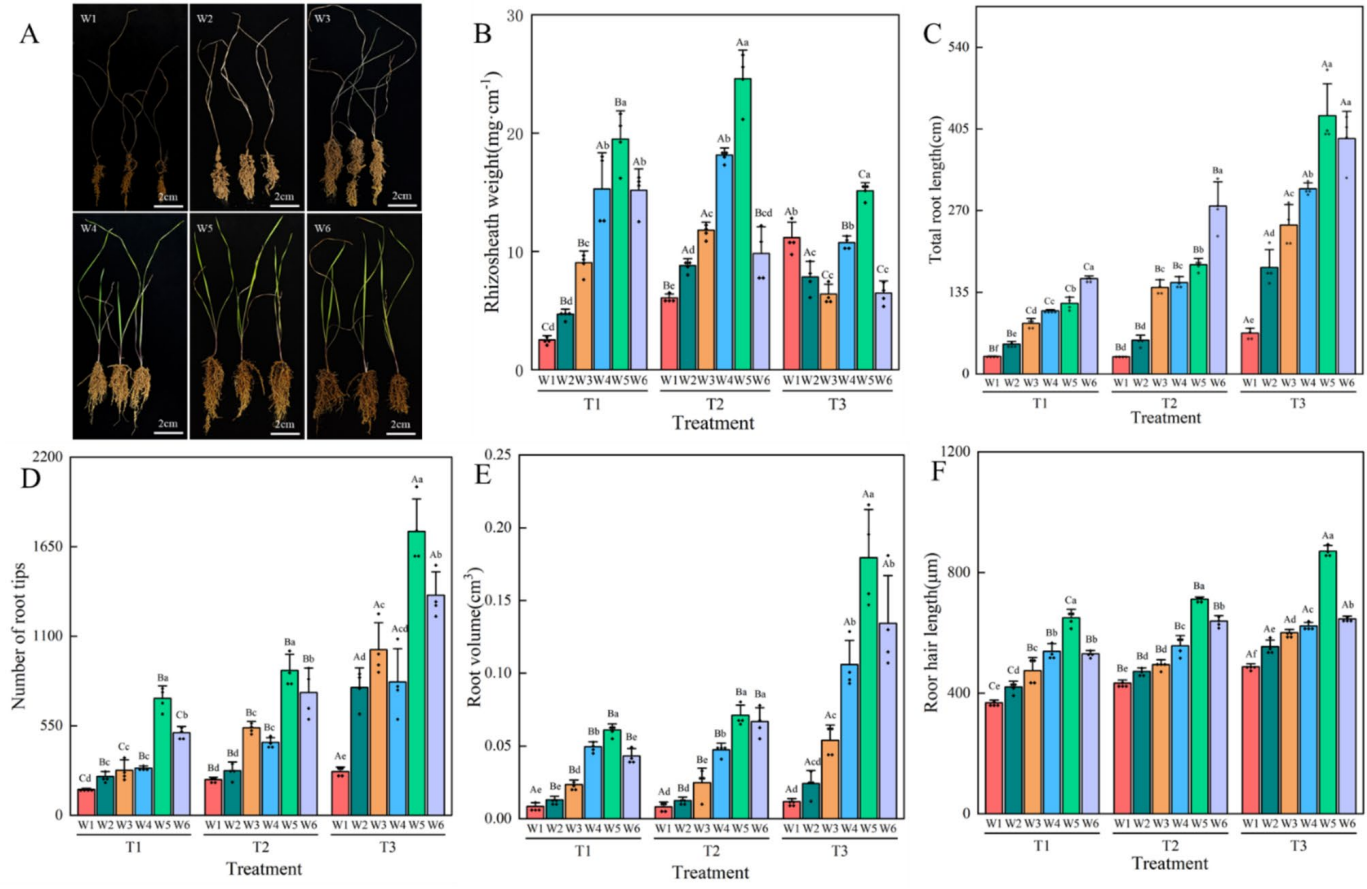
Root morphogenesis and rhizosheath formation in *K. hirsuta* seedlings under varying water regimes. Note: In Fig. 2A, W1, W2, and W3 represent re-watered to 10%, 25%, 40%FC, respectively. W4, W5, and W6 represent maintained at 10%, 25%, 40%FC, respectively; The different lowercase letters in Fig. 2B-I indicate significant differences (*P*<0.05) in different moisture gradients during the same period; Different capital letters indicate significant differences (*P*<0.05) at different stages under the same moisture gradient, with T1, T2, and T3 representing stages 1, 2, and 3, respectively. The same below.

As shown in Fig. 2A/B, rhizosheath biomass responded non-linearly to increasing water stress. Under maintained 25% FC (W5), rhizosheath dry weight reached its maximum at T1 and T2 (19.49 mg/cm and 24.60 mg/cm, respectively)—6.7 and 3.04 times greater, respectively, than under the W1 treatment (drought–rehydration at 10%; *P* < 0.05). At T3, the W5 treatment maintained the highest rhizosheath accumulation (15.13 mg/cm), whereas values under W2, W3, and W6 were significantly lower than in other groups (*P* < 0.05). The applied water treatments induced clear temporal shifts in rhizosheath development. Under W1, rhizosheath weight increased continuously across growth stages, reaching 11.18 mg/cm at T3—a 341.9% increase relative to T1 (*P* < 0.05). In contrast, under W5, rhizosheath weight peaked at T2 (24.60 mg/cm) and decreased by 38.5% by T3, yet remained significantly higher than all other treatments at that stage (*P* < 0.05). Under W6, rhizosheath weight declined linearly over time, falling by 57.3% at T3 compared to T1 (*P* < 0.05*)*. Overall, the W5 treatment markedly promoted rhizosheath accumulation, likely by enhancing root exudation and stimulating microbial activity. Notably, re‑watered to 10% FC (W1) activated rhizosheath formation in later growth (T3) through a compensatory growth response. Conversely, higher initial soil moisture (W6) appeared to reduce root stress perception, thereby the rhizosheath declines.

#### Adaptive responses of root architecture and root hair traits to water stress

Graded water stress induced coordinated alterations in both root architecture and root hair traits of *K. hirsuta* (Fig. 2C–F). Under the 25% FC (W5) treatment, plants developed an integrated drought-resistance strategy. At the architectural level, total root length increased linearly with rising soil moisture gradient during the T1 and T2 periods (*P* < 0.05; Fig. 2C). The W5 treatment markedly promoted root branching, resulting in 5.48‑fold and 6.93‑fold increases in root tip number and branch number, respectively, compared to the W1 treatment by T3 (*P* < 0.05; Fig. 2D, Supplementary Figure 1B). This indicates that moderate drought stress stimulated the formation of a complex branching network, thereby enhancing soil exploration. Similarly, root surface area and root volume also peaked under the W5 treatment, exceeding those in W1 by 9.02‑fold and 15.28‑fold, respectively (*P* < 0.05; Fig. 2E, Supplementary Figure 1A).

As shown in Fig. 2F, different water treatments had a significant impact on the root hair characteristics of *K. hirsuta*. Overall, both root hair length and root hair density exhibited a trend of initially increasing and then decreasing across the water level. Regarding root hair length (Fig. 2F), values across all growth stages followed T3 > T2 > T1 (*P* < 0.05). The W5 treatment resulted in the greatest root hair length, measuring 650.29 μm, 712.10 μm, and 871.03 μm at the T1, T2, and T3 stages, respectively (*P* < 0.05). For root hair density (Supplementary Figure 1C), the W5 treatment yielded the highest density during the T2 and T3 stages.

### Variation characteristics of AM fungal colonization in *K. hirsuta*

#### Characteristics of colonization changes of AM fungi

**Fig. 3:**
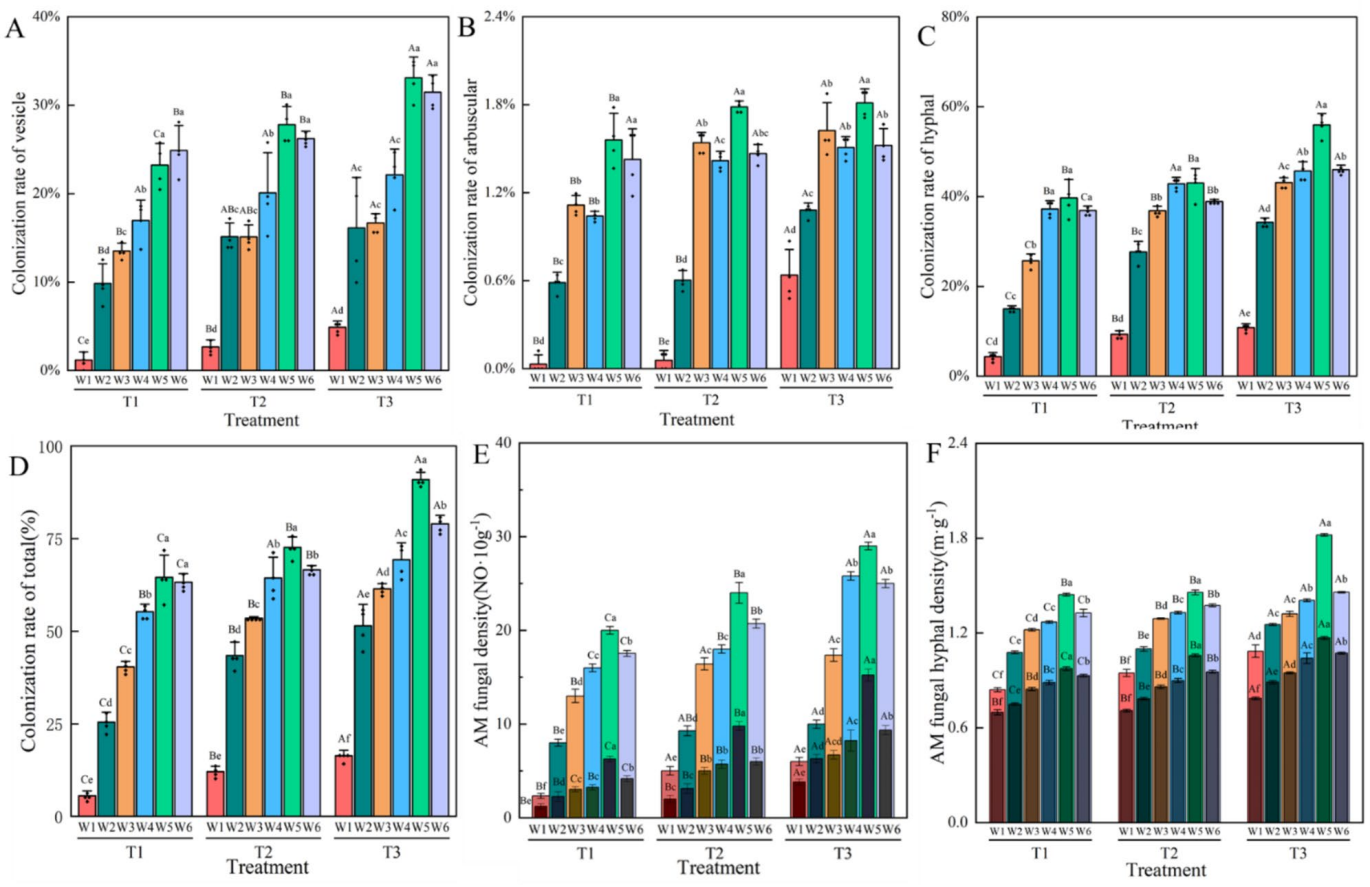
Colonisation and propagule distribution of AMF at different growth stages under water stress. Note: In Fig. 3E, F, the light-colored areas in the diagram represent rhizosheath soil, while the dark-colored areas indicate root zone soil.

The colonisation dynamics of arbuscular mycorrhizal (AM) fungi showed significant variation (Fig. 3). Total colonisation rate increased progressively across growth stages, from 41.51% at T1 to 61.40% at T3 (Fig. 3D), and was highest under sustained 25% water stress (W5; *P* < 0.05). Vesicle colonisation rates (ranging from 1.18% to 33.10%) were significantly higher under W5 and W6 than in other treatments (*P* < 0.05; Fig. 3A), suggesting that relatively high moisture conditions (≥25%) favour the formation of these storage structures. The arbuscule colonisation rate showed greater fluctuation (Fig. 3B), with W5 maintaining the highest values throughout T1–T3 (1.56–1.81%). The synchronisation of arbuscule development with rhizosheath formation points to a potential role in rhizosheath construction through enhanced carbon–water exchange. Hyphal colonisation under W5 exhibited a stress-adaptation pattern (Fig. 3C), increasing by 30.08–40.89% at T3 compared to T1/T2. This supports the view that hyphal network expansion is a key mechanism in AM–host interaction during mid-to-late drought stages. Notably, total colonisation under W5 exceeded 90% at T3, significantly surpassing all other treatments (*P* < 0.05). This indicates that 25% water stress effectively promotes a functional AM–plant symbiosis by improving hyphal colonisation efficiency and host resource allocation.

#### Distribution characteristics of AM fungal spores and hyphae

The spatial distribution of AM fungal propagules and hyphal networks was significantly influenced by soil water regime (Fig. 3E, F). Sustained 25% water stress (W5) markedly enhanced both spore and hyphal densities in the rhizosheath (WR) and rhizosphere (WS) soils. By T3, under W5, spore density reached 29 spores/10 g and hyphal density 1.82 m/g in the WR zone, while values in the WS zone were 14.5 spores/10 g and 1.17 m/g. These represent increases of 3.83‑fold, 0.68‑fold, 3.14‑fold, and 0.49‑fold, respectively, compared to the W1 treatment (*P *< 0.05). Over time, AM fungal biomass in both WR and WS zones accumulated progressively. Under W5, WR spore density increased by 45% from T1 to T3, and hyphal density by 26.39% (*P *< 0.05)—significantly greater than under other treatments. Biomass was consistently higher in the WR than in the WS zone, indicating that the rhizosheath acts as a hotspot for AM fungal enrichment, likely due to root exudates and physical retention. These results suggest that 25% water stress promotes spore germination and hyphal network expansion by optimising soil moisture and host carbon supply. This functional coupling is reflected in the total colonisation rate of 90.88% at T3 (Fig. 3D), supporting a coordinated rhizosheath–AMF drought adaptation strategy.

#### Principal component analysis reveals multidimensional root adaptation to water stress

**Fig. 4:**
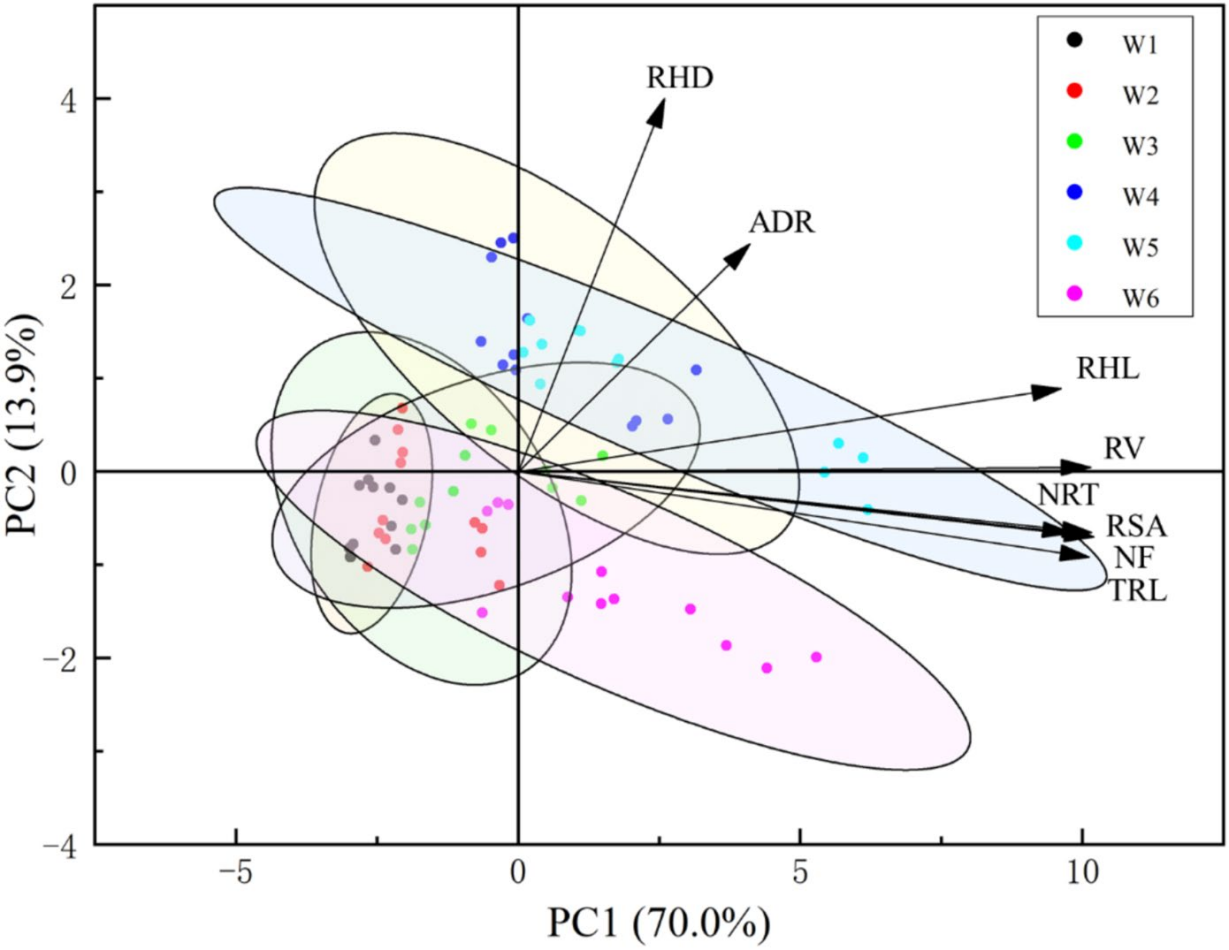
Multidimensional characterization of root architectural traits: Drought adaptation strategies revealed by principal component analysis. Note: TRL represents total root length, RSA represents root surface area, ARD represents average root diameter, RV represents root volume, NRT represents number of root tips, NF represents number of forks, RHL represents root hair length, RHD represents root hair density. respectively. The same below.

Principal component analysis (PCA) of root phenotypic traits indicated that *K. hirsuta* responds to water stress through multidimensional morphological strategies (Fig. 4). The first two principal components (PC1 and PC2) together explained 83.93% of the total phenotypic variation, with PC1 accounting for 70.02% (λ = 5.60) and PC2 for 13.91% (λ = 1.11). PC1 was strongly associated with total root length (loading: 0.404), total root surface area (0.406), total root volume (0.406), and number of branches (0.408), reflecting the spatial expansion capacity of the root system. PC2 was closely linked to root hair density (0.80) and average root diameter (0.49), representing adaptation to heterogeneous soil microenvironments. Notably, the W5 treatment exhibited a pronounced distribution along both PC1 and PC2 axes. Under this regime, plants synergistically enhanced branching complexity and root hair density, achieving a functional integration of wide-ranging exploration and local optimisation. These findings support the hypothesis that 25% water stress reshapes both root topological structure and fine-scale phenotypic traits, forming a hierarchical drought adaptation network. Together with the rhizosheath–AMF interaction system, this network elucidates the integrated adaptive strategy employed by plants in sandy habitats.

#### Biomass allocation shifts in response to water stress gradient

**Fig. 5:**
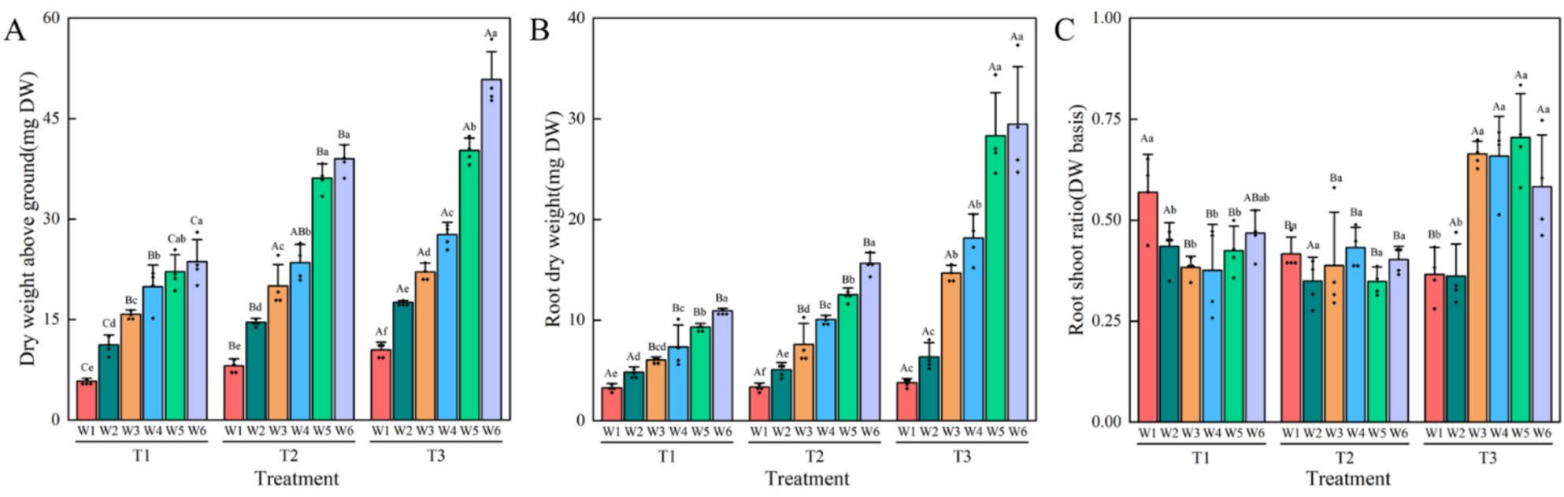
Biomass allocation and dynamic regulation of root cap.

Biomass and root-to-shoot ratio (R/S) of *K. hirsuta* exhibited distinct variations under different water treatments. Overall, total plant biomass increased progressively throughout the growth periods (from T1 to T3). Within the same period, shoot fresh weight, shoot dry weight, and root dry weight increased significantly with rising soil moisture gradient (10%–40% FC) (*P* < 0.05; Supplementary Figure 2A, Fig. 5A, B). The response of root biomass to water availability was more complex. Root fresh weight did not increase linearly with higher moisture (Supplementary Figure 2B); instead, it showed a pattern of initial increase followed by a decrease across T1 to T3, peaking under the W5 treatment. At T2 and T3, root fresh weight in W5 was significantly higher than in other treatments (*P* < 0.05).

Changes in R/S further reflected dynamic adjustments in plant resource allocation strategies in response to water regimes. At the early stage (T1), R/S first decreased and then increased along the moisture gradient, with the highest value observed in W1. By the mid-stage (T2), no significant differences were detected among treatments. At the late stage (T3), moderate- to high-moisture treatments (W3–W6) had significantly higher R/S than severe drought treatments (W1–W2) (*P* < 0.05). Over time, R/S exhibited divergent trends among treatments: it gradually declined under severe drought (W1-W2), increased steadily under low to moderate moisture (W3-W4), and showed an initial decrease followed by an increase under moderate to high moisture (W5-W6, Fig. 5C).

#### Functional analysis of the AMF–plant interaction network in drought resistance

**Fig. 6:**
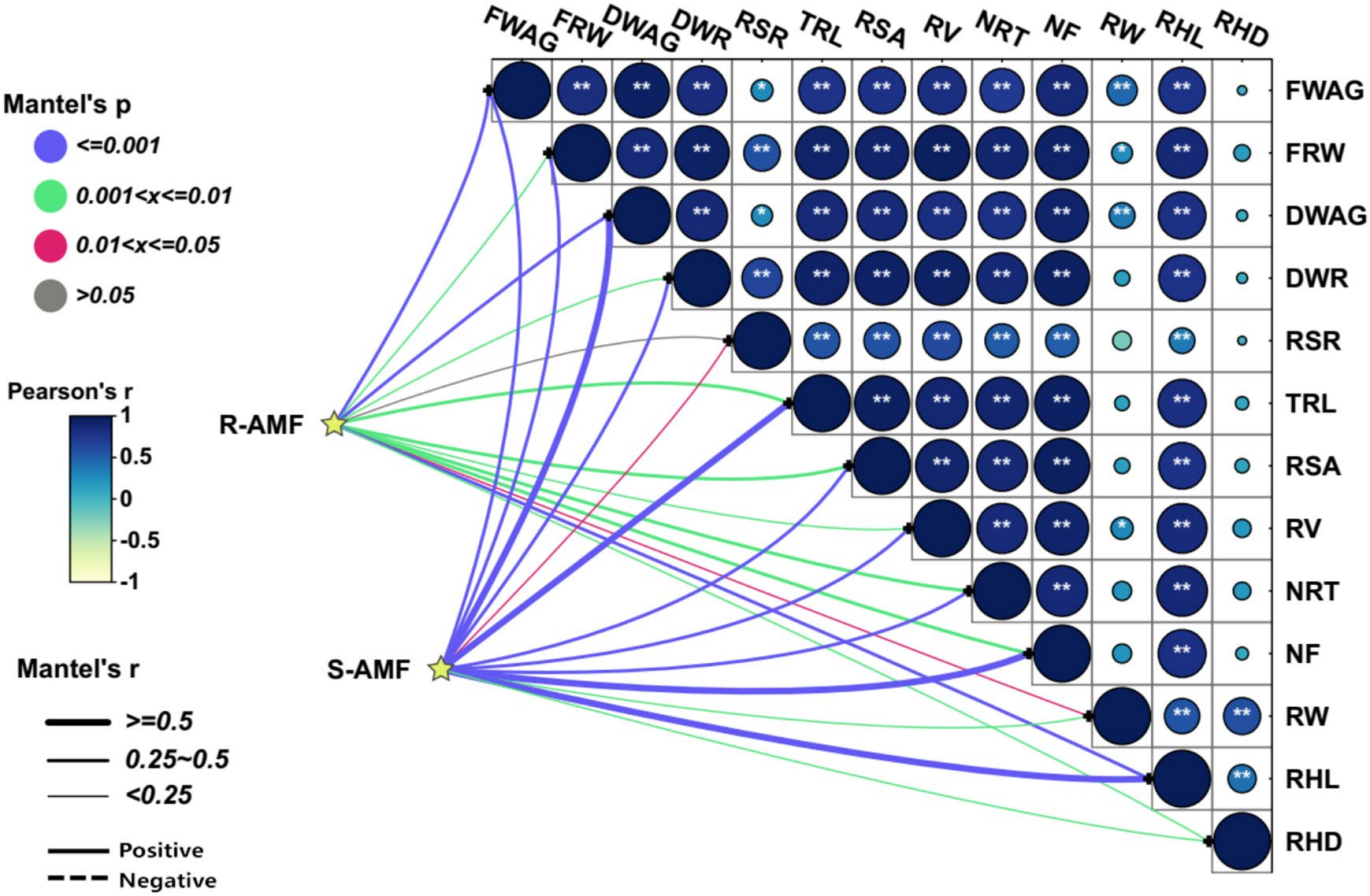
Functional integration of AMF-plant phenotypic interaction networks via Mantel test. Note: S-AMF in the figure represents soil-borne arbuscular mycorrhizal fungi, R-AMF represents root-colonising arbuscular mycorrhizal fungi, FWAG represents aboveground fresh weight, FRW represents root fresh weight, DWAG represents aboveground dry weight, DWR represents root dry weight, RSR represents root–shoot ratio. RW represents rhizosheath weight

Mantel tests revealed a highly significant correlation (*P* < 0.01) between soil-borne arbuscular mycorrhizal fungi (S-AMF) and multiple biomass traits in *K. hirsuta* (Fig. 6). S-AMF abundance was significant positive correlation with aboveground fresh weight (FWAG), root fresh weight (FRW), aboveground dry weight (DWAG), root dry weight (DWR), root–shoot ratio (RSR), and key root morphological traits—including total root length (TRL), root surface area (RSA), root volume (RV), number of root tips (NRT), and number of forks (NF). Rhizosheath-related traits such as rhizosheath weight (RW), root hair length (RHL), and root hair density (RHD) were also significantly correlated with S-AMF. These results suggest that S-AMF enhance plant drought resistance by expanding the root–soil interface and facilitating efficient carbon–phosphorus exchange. In comparison, root-colonising AMF (R-AMF) showed significant positive correlations (*P* < 0.05) with all traits except RSR. Aboveground and root fresh weights (FWAG and FRW) exhibited highly consistent variation, displaying strong positive correlations (*P* < 0.01) with most biomass, architectural, and root hair traits, and a significant correlation with RSR (*P* < 0.05). Dry weight traits (DWAG and DWR) were significantly correlated (*P* < 0.05) with TRL, RSA, RV, NRT, NF, and RHL, and showed a highly significant positive correlation (*P* < 0.01) with RW. Within the root system, strong interdependencies (*P* < 0.01) were observed among architectural traits (TRL, RSA, RV, NRT, NF). Root volume was also significantly correlated with rhizosheath weight (*P* < 0.05), and tightly coupled relationships (*P* < 0.01) linked RW, RHL, and RHD.

#### Structural equation modelling reveals hierarchical regulation of rhizosheath formation

**Fig. 7:**
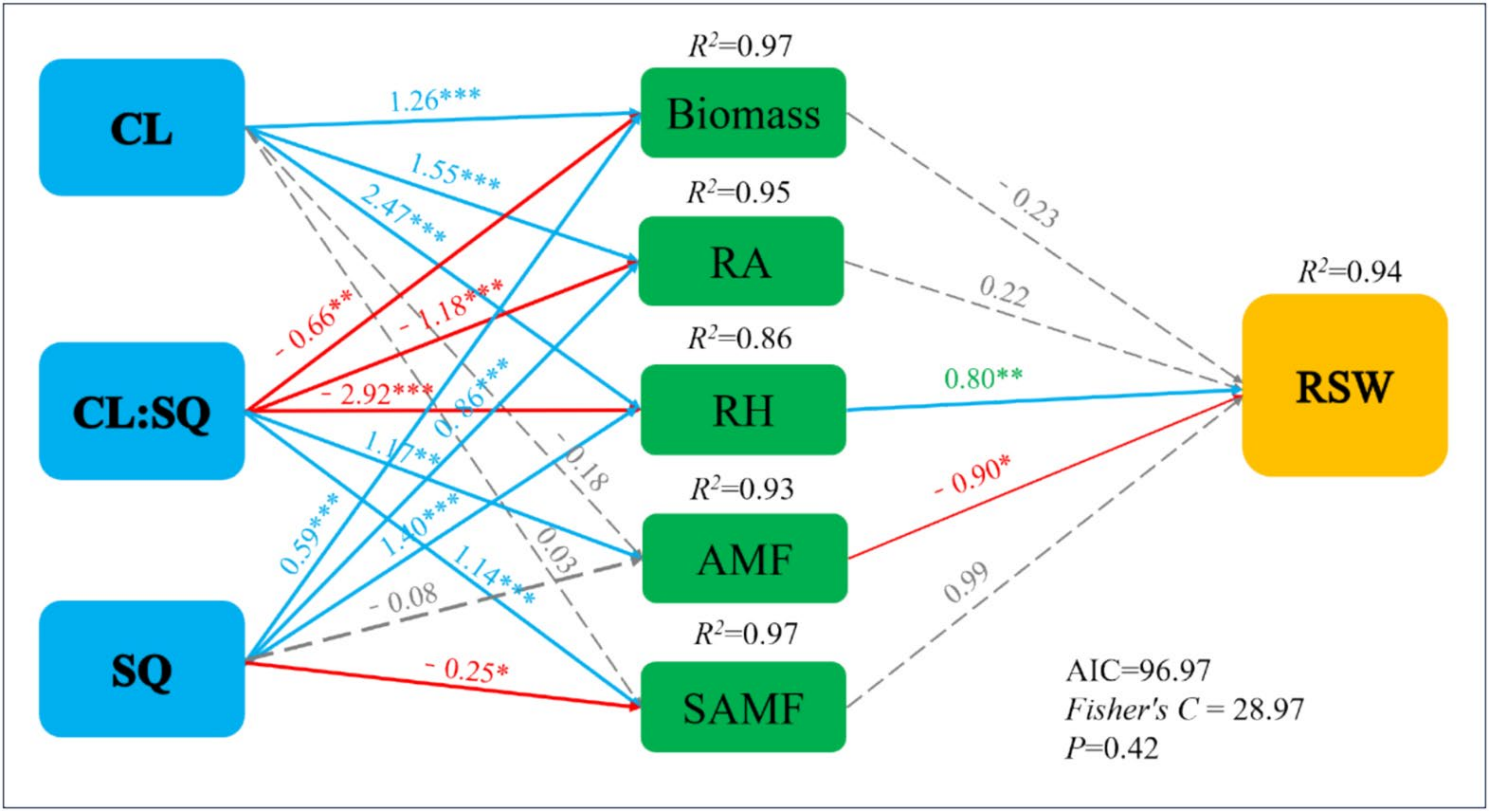
Multifactorial pathways driving rhizosheath formation. Note: CL in the figure represents different water treatments, SQ represents different periods, CL: SQ represents the interaction between treatments and periods, and Biomass represents biomass (aboveground fresh weight, underground fresh weight, aboveground dry weight, underground dry weight, root shoot ratio); RA represents root morphology (total root length, root surface area, average root diameter, root volume, number of root tips, number of bifurcations); RH represents root hair characteristics (root hair length, root hair density); AMF represents the infection rate of arbuscular mycorrhizal fungi (vesicle infection rate, arbuscular infection rate, hyphal infection rate, and total infection rate); SAMF represents the infection rate of arbuscular mycorrhizal fungi (AMF hyphae, spores) in rhizosheath soil; RSW represents the weight of rhizosheath soil.

A segmented structural equation model (SEM) was used to elucidate how drought stress and growth stage interact to regulate rhizosheath formation, revealing a hierarchical regulatory pathway (Fig. 7). The model demonstrated good fit (Fisher’s C = 28.97, P = 0.42). Root hair traits (path coefficient = 0.80) and AM fungal colonisation rate (–0.90) exerted the strongest direct effects on rhizosheath formation, while biomass allocation (indirect effect = 1.26), root architectural plasticity (1.55), and AM fungal activity in rhizosheath soil (1.14) functioned through indirect pathways. Growth stage progression significantly enhanced the positive effects of root architecture (0.86) and root hair plasticity (1.40) on rhizosheath development, but suppressed soil AM fungal activity (–0.25), indicating a stage-dependent resource trade-off in plant–microbe interactions. Maintained at 25% FC (W5) strengthened the synergistic contribution of root hair traits (–2.92) and AM fungal colonisation (1.17) to rhizosheath formation via treatment–stage interaction, highlighting its role in modulating development through the mycorrhizal network. AM fungal colonisation rate (total effect: –0.90) balanced root carbon expenditure via negative regulation, whereas root hair traits (total effect: 0.80) served as physical structures expanding the root–fungus interface. Together, these form a complementary mechanism of “hyphal synergy and morphological adaptation.” The model indicates that rhizosheath formation arises from combined contributions of plant phenotypic plasticity (54.3%) and microbial interaction networks (45.7%). Under experimental conditions, the W5 treatment promoted functional assembly of rhizosheath modules in sandy habitats by optimising dynamic equilibrium among root hairs, AMF, and the soil environment. These findings offer a theoretical framework for understanding ecological and evolutionary mechanisms in plant–microbe drought adaptation.

## Discussion

### Adaptive mechanism of root morphological plasticity to alternate drought–rehydration

Root architectural plasticity enables plants to efficiently explore and acquire soil resources, a trait of considerable ecological importance in arid habitats (Fig. 2). Unlike the inhibitory effect of drought on root phenotypes in foxtail millet (*Setaria italica*) (Zhao et al.,^20^), this study found that *K. hirsuta* significantly increased total root length (by 5.48-fold at stage T3 compared to W1), root surface area (8.02-fold), and branch number (6.93-fold) under 25–40% moisture gradients. This difference may reflect evolutionary adaptations of *K. hirsuta* as a pioneer species in desertified grasslands: sandy soils maintain adequate pore oxygen even at 25–40% moisture, satisfying root aerobic metabolism. As shown in prior studies, *K. hirsuta* enhances deep soil water acquisition while optimising resource capture in surface microzones by increasing root branching angles and root hair density. Notably, it is noteworthy that root volume consistently exhibited an initial increase followed by a decrease across different periods, reaching its maximum under the W5 treatment. Meanwhile, the number of root tips showed periodic fluctuations during the T2 and T3 stages. This pattern suggests that plants dynamically regulate the biomass allocation between storage roots and absorptive roots to balance short-term survival and long-term adaptation needs^21^. In contrast to the root expansion of perennial ryegrass (*Lolium perenne*) under ample moisture^22^, *K. hirsuta* under sustained 40% moisture (W6) showed increased total root length (389.06 cm at T3) and lower AM fungal colonization, this suggests that mild water stress may disrupt the synergistic relationship between root morphological plasticity and microbial interactions. This “water paradox” phenomenon indicates that moderate drought (25% of field capacity) may activate ABA signaling-mediated reprogramming of root architecture, shifting the branching pattern from quantitative expansion toward quality optimization^23,24^. This process forms a positive feedback loop with the establishment of mycorrhizal symbiotic networks, jointly supporting the construction of the rhizosheath’s ecological functions^25^. The findings provide a novel theoretical perspective for understanding the ecological and evolutionary mechanisms underlying root plasticity in psammophytes.

Root hairs, as frontline sensors of soil heterogeneity, exhibit phenotypic plasticity in length and density, a key strategy for adapting to moisture variation. Under maintained at 25% FC (W5), root hair length increased significantly (871.03 μm at T3, 34.78% longer than W6, *P* < 0.05) while maintaining high density (66 root hairs/mm), forming a "super surface area" absorption mode similar to Japanese brome (*Bromus japonicus*)^26^. However, *K. hirsuta* shows unique spatiotemporal regulation: root hair length increased continuously through growth stages (T1→T3: 650.29→871.03 μm), while density peaked at T2 then declined, suggesting that mature plants extend the lifespan of existing root hairs rather than producing new ones. This contrasts with the "quantity–quality" trade-off in typical dicot root hair development^27^. The 40% moisture treatment (W6) inhibited root hair density, and drought–rehydration (W1) impeded both length and density, indicating that moderate sustained drought may activate ethylene-mediated root hair elongation^28^, while high or fluctuating moisture disrupts calcium oscillation signalling, leading to phenotypic imbalance^29^. The strong synergy among root hair length, AM fungal hyphal colonization, and rhizosheath biomass (Fig. 5) suggests root hairs act not only as physical absorption organs but also as chemical signalling hubs for microbial interaction^30^. Flavonoids and other signals secreted from root hair tips may guide hyphal growth via chemotaxis^31^. These results support the "root hair–hyphal synergy" theory and indicate that psammophytes have evolved a unique rhizosheath construction strategy integrating phenotypic plasticity and microbial cooperation, offering biomimetic inspiration for drought-resistant crop root design^25^.

#### Regulatory mechanism of moisture threshold on the AMF–plant symbiotic relationship

The intensity of AM fungal symbiosis with host plants is non-linearly regulated by moisture and exhibits species and habitat specificity (Fig. 2). AM fungi form symbiotic associations with most terrestrial plants^32^ and enhance host stress resistance, competitive ability, and survival, thereby influencing plant community structure and ecosystem productivity^33,34^.

This study demonstrates that the total AMF colonization rate in *K. hirsuta* increased significantly over time and as stress diminished, peaking under maintained at 25% FC (W5). This pattern is largely consistent with the “drought‑promoted” symbiotic mode observed in grassland plants such as *Agropyron cristatum*^35^, which may explain the lower AMF colonization under the 10% rewatering treatment (W1). Such divergence likely stems from the evolutionary adaptation of psammophytes: the high permeability of sandy soil maintains sufficient pore‑space oxygen concentration even under 25%–40% moisture gradients, meeting the aerobic metabolic demands of AMF. Moderate drought (25%) may activate the JA signaling pathway, promoting root exudation of strigolactones and other signal molecules that specifically attract AMF hyphal colonization^36^. Although the 40% moisture treatment (W6) sustained a relatively high colonization rate (78.99%), its hyphal network density (1.46 m·g⁻^1^) was significantly lower than that under W5 (*P* < 0.05), suggesting that mild water stress might inhibit functional differentiation of AMF, delaying arbuscule formation or reducing lipid metabolism^37,38^. Notably, rewatering treatments (W1–W3) reduced AMF colonization by 21.8–38.5% compared with the sustained‑drought group (*P* < 0.05), which could be attributed to the role of AMF in enhancing plant antioxidant capacity through increased proline and reduced glutathione consumption, thereby mitigating drought damage,however, under severe drought, an imbalanced antioxidant system may indirectly lead to colonization failure^37–39^. AMF colonization exerted a direct and dominant feedback regulatory effect on rhizosheath formation, as revealed by a structural equation modeling path coefficient of −0.91. This negative coefficient does not indicate direct inhibition of rhizosheath formation by AMF, but rather quantifies the dynamic trade-off process mediated by microbial interactions within the rhizosheath microenvironment. The rhizosheath functions as an edaphic "mini-oasis"^40^, where limited space and resources inevitably intensify competition between non-AMF microorganisms and AMF, thereby exerting competitive feedback inhibition on AMF colonization efficiency^41,42^. The negative pathway captured by the structural equation model thus serves as a quantitative representation of this competition-driven feedback regulatory mechanism. The synchronous peak values of rhizosheath biomass and AMF colonization rate under the W5 treatment provide strong evidence for the ecological plasticity of this feedback regulation. Specifically, under optimal water conditions (25% FC), optimized root morphology significantly enhanced rhizosheath resource supply capacity, effectively alleviating microbial competition and shifting the relationship between AMF colonization and rhizosheath accumulation from mutual constraint under resource limitation to positive synergy under environmental optimization. Collectively, these findings establish the negative path coefficient of AMF colonization as a core regulatory signal maintaining the dynamic equilibrium of the psammophyte-AMF symbiotic system. This coefficient simultaneously quantifies microbial competition trade-offs under resource constraints and aligns coherently with the positive association between AMF and rhizosheath formation under optimal conditions, thereby providing both theoretical support and practical insights for mycorrhiza-based ecological restoration in degraded grasslands^43^.

#### Analysis of rhizosheath formation in K. hirsuta under drought and rehydration conditions

From a physio-ecological perspective, the rhizosheath plays multiple roles in drought adaptation. On one hand, it enhances soil aggregate stability by accumulating root exudates (e.g., polysaccharides and mucilage), thereby slowing water loss in the rhizosphere and maintaining intimate root–soil contact under water stress, which reduces hydraulic resistance^25^. On the other hand, rhizosheath formation is closely associated with root hair development. Root hairs act as a physical scaffold for the rhizosheath,under drought conditions they expand the diffusion range of exudates and promote soil particle adhesion, leading to the formation of a thicker and more stable rhizosheath layer^16,44^. In this study, root hair structural traits and AMF colonization rate were also observed to directly and significantly influence rhizosheath formation, consistent with previous reports on the synergistic roles of root hairs and microorganisms in promoting rhizosheath development^7^. Furthermore, rhizosheath formation and function are profoundly regulated by the rhizosphere microbial community. Under drought, specific microbial taxa (e.g., Actinobacteria) become significantly enriched within the rhizosheath. Beneficial microbes such as *Streptomyces* can indirectly stabilize rhizosheath structure by promoting root growth and secreting adhesive substances (Santos‑Medellín et al.,^44^). This study further indicates that rhizosheath formation is influenced by multiple factors,in addition to root hairs and AMF, root mucilage concentration, metabolite profiles, and microbial community structure may all participate in regulating rhizosheath development, warranting further systematic investigation.

On the Qinghai–Tibet Plateau, where climate change is intensifying and drought events are becoming more frequent, plants in their natural habitats experience prolonged and severe drought. Under such conditions, rhizosheath formation plays a crucial ecological role in maintaining the function of fragile ecosystems^45^. This study reveals that the rhizosheath integrates physical protection, microbial symbiosis, and root morphological plasticity to form a multi‑dimensional adaptation network, significantly enhancing the survival capacity of *K. hirsuta* at the three‑leaf stage in fluctuating moisture environments. Under 25% field capacity (FC) stress, the rhizosheath not only directly reduces water evaporation and enhances nutrient retention (El‑Keblawy et al.,^46^) but also synergistically improves water and nutrient acquisition efficiency by promoting arbuscular mycorrhizal fungi (AMF) colonization^47^. Moreover, rhizosheath formation and its associated root activities help stabilize sandy soil structure and increase resistance to wind erosion, thereby slowing grassland degradation. As a functional nexus in alpine desertified grassland ecosystems, the rhizosheath supports ecological restoration and stability. Its underlying mechanisms provide a key theoretical basis for selecting stress‑tolerant grass species and rehabilitating degraded ecosystems^30,46^).

## Conclusion

This study demonstrates that *K. hirsuta* adapts to fluctuating water conditions through the coordinated regulation of root system architecture, biomass allocation, and symbiotic association with arbuscular mycorrhizal fungi (AMF), which collectively enhance rhizosheath formation. Under maintained 25% FC (W5), root surface area, volume, root hair length, and root hair density all reached peak values, while root branching complexity was increased to improve water stress adaptation. Under this condition, rhizosheath biomass accumulation, root morphological development, and AMF colonization were significantly enhanced. Structural equation modeling further identified the extent of AMF colonization and root hair traits as key regulators of rhizosheath formation. Notably, the negative direct effect exhibited by AMF colonization does not indicate an inhibitory role, but rather quantifies the intensity of feedback regulation mediated by microbial competition within the rhizosheath microenvironment, reflecting the carbon allocation trade-off mechanism of the plant-fungal symbiosis under resource constraints. These factors interact by coordinating biomass allocation, modulating root architecture, and modifying the soil microenvironment, thereby forming a multidimensional interactive adaptation network. These findings systematically elucidate the ecophysiological mechanisms by which plant–microbe interactions promote rhizosheath formation under varying water regimes, providing a theoretical basis for selecting stress‑tolerant grass species to restore degraded grassland ecosystems.

## Supplementary Information

Supplementary Information.

## Data Availability

Data will be available upon request from the corresponding author.
