# Supplementary material for: Mechanisms underlying rhizosheath dynamics in Kengyilia hirsuta in response to alternating drought and rewatering
Source: Sci Rep. 2026 Apr 24;16:19077. doi: 10.1038/s41598-026-49036-7 (PMC13280164; doi:10.1038/s41598-026-49036-7)
Supplement: Supplementary file 1 — Supplementary Information. [file 41598_2026_49036_MOESM1_ESM.docx]

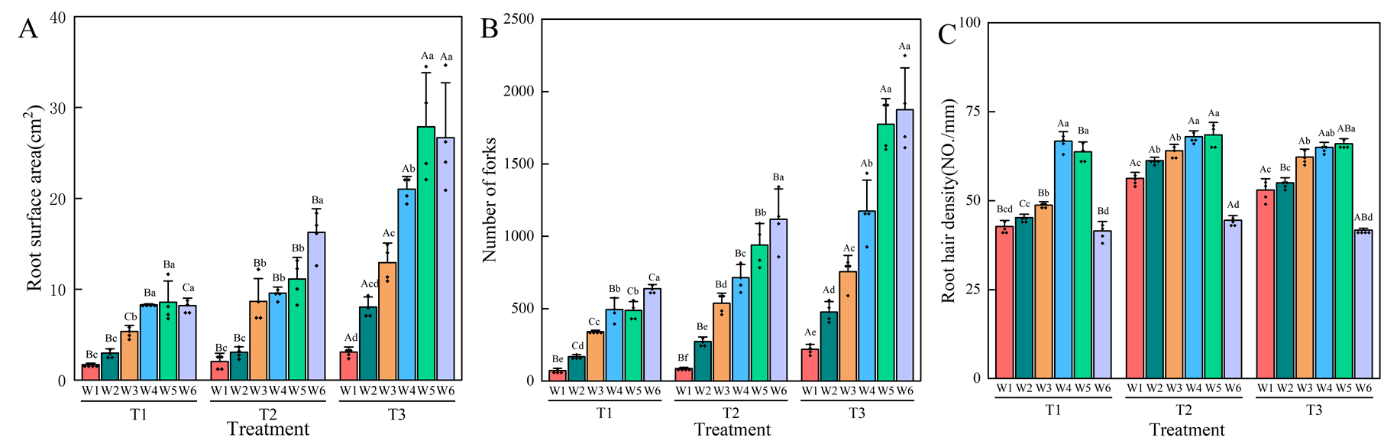


Supplementary Figure 1 Characteristics of changes in root surface area, number of root forks and root hair density of *K. hirsuta* under different water treatments


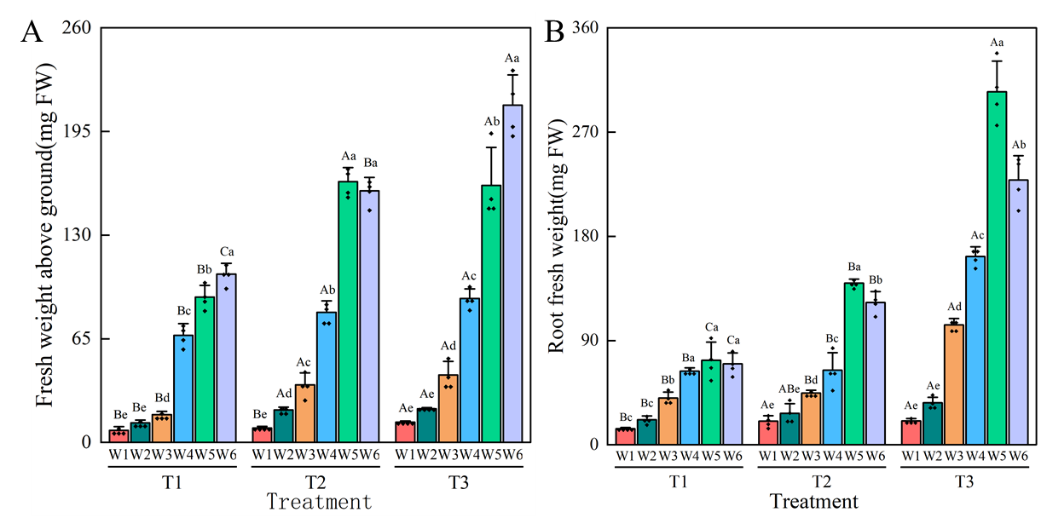


Supplementary Figure 2 Characteristics of fresh weight changes in *K. hirsuta* under different moisture treatments
